# Usefulness of four-dimensional noise reduction filtering using a similarity algorithm in low-dose dynamic computed tomography for the evaluation of breast cancer: a preliminary study

**DOI:** 10.1007/s11604-024-01730-0

**Published:** 2025-01-24

**Authors:** Daichi Uraoka, Megumi Matsuda, Yuki Tanabe, Naoto Kawaguchi, Chihiro Nishiyama, Ayaka Okada, Koichiro Uda, Hiroshi Suekuni, Hikaru Nishiyama, Yoshiaki Kamei, Mie Kurata, Riko Kitazawa, Shota Nakano, Teruhito Kido

**Affiliations:** 1 Department of Radiology, Ehime University Graduate School of Medicine, Shitsukawa, Toon, Ehime 791-0295 Japan; 2 Department of Radiology, Ehime Prefectural Central Hospital, Kasugamachi, Matsuyama, Ehime 790-0024 Japan; 3 Department of Radiology, Ehime University Hospital, Shitsukawa, Toon, Ehime 791-0295 Japan; 4 Breast Center, Ehime University Hospital, Shitsukawa, Toon, Ehime 791-0295 Japan; 5 Department of Pathology, Ehime University Proteo-Science Center, Shitsukawa, Toon, Ehime 791-0295 Japan; 6 Department of Analytical Pathology, Ehime University Graduate School of Medicine, Shitsukawa, Toon, Ehime 791-0295 Japan; 7 Division of Diagnostic Pathology, Ehime University Hospital, Shitsukawa, Toon, Ehime 791-0295 Japan; 8 Canon Medical Systems Corporation, Shimoishigami, Otawara, Tochigi 324-8550 Japan

**Keywords:** Breast cancer, Computed tomography, Four-dimensional noise reduction filtering using a similarity algorithm, Radiation dose reduction

## Abstract

**Purpose:**

To evaluate the effects of four-dimensional noise reduction filtering using a similarity algorithm (4D-SF) on the image quality and tumor visibility of low-dose dynamic computed tomography (CT) in evaluating breast cancer.

**Materials and methods:**

Thirty-four patients with 38 lesions who underwent low-dose dynamic breast CT and were pathologically diagnosed with breast cancer were enrolled. Dynamic CT images were reconstructed using iterative reconstruction alone or in combination with 4D-SF. We selected the peak enhancement phase image of breast cancer for each patient for quantitative and qualitative evaluations of image quality and measurement of the maximum diameter of breast cancer. The signal-to-noise and contrast-to-noise ratios were calculated for quantitative evaluation. The maximum diameters of the breast cancer were measured from the images obtained with and without 4D-SF (4D-SF ±) (size-4D-SF + and size-4D-SF-) and for the pathological specimen (size-PS) and compared.

**Results:**

The median and interquartile ranges of the signal-to-noise ratio [4D-SF-: 3.03 (2.54–4.17) vs 4D-SF + : 5.52 (4.75–6.66)] and contrast-to-noise ratio [4D-SF-: 2.88 (2.00–3.60) vs 4D-SF + : 7.84 (4.65–10.35)] were significantly higher for 4D-SF + than for 4D-SF- (p < 0.001). The overall image quality (Observer 1, p < 0.001; Observer 2, p < 0.001) and tumor margin sharpness scores (Observer 1, p = 0.003; Observer 2, p < 0.001) were significantly higher for 4D-SF + than for 4D-SF-. The tumor contrast scores for 4D-SF + and 4D-SF- were not significantly different (Observers 1, 2; p = 0.083). Size-4D-SF- was significantly smaller than size-PS (p < 0.001); size-4D-SF + was also smaller than size-PS, but the difference was not significant (p = 0.088). The Spearman’s rank correlation coefficient was 0.65 for size-PS and size-4D-SF- and 0.77 for size-PS and size-4D-SF + .

**Conclusion:**

The 4D-SF can improve the image quality and tumor visibility of low-dose dynamic CT in evaluating breast cancer extent due to noise reduction.

## Introduction

Female breast cancer had the second highest global cancer incidence in 2022 [1]. In addition to mastectomy, breast-conserving surgery is well-established for its treatment [2, 3]. Precise evaluation of tumor size and extent of breast cancer is crucial for determining the optimal surgical procedure and minimizing the risk of local recurrence after surgery.

Contrast-enhanced magnetic resonance imaging (CE-MRI) is considered the most accurate imaging modality for the detection and evaluation of breast cancer [4–7]. Contrast-enhanced computed tomography (CE-CT) is considered inferior to CE-MRI for the detection and diagnosis of breast cancer. In addition, CT is a major source of radiation exposure [6, 8, 9]. However, CE-CT has some advantages over CE-MRI, such as shorter scan time, supine scanning position (which is the same for the surgery), and the ability to obtain whole-body images for cancer staging [6]. Moreover, MRI can be problematic for patients with claustrophobia or those with metallic implants.

The peak enhancement time for breast cancer on CE-CT varies across reports, and the optimal scan timing for the evaluation of breast cancer extent, including the intraductal components, has not been established [10–13]. The increase in radiation exposure is a major problem, but dynamic imaging may be preferable for evaluating the extent of breast cancer more accurately.

The iterative reconstruction (IR) technique is widely used to reduce radiation exposure without impairing diagnostic image quality. However, the IR techniques operate in 3D space and filter in the spatial domain. As neighboring voxels do not need to correspond to the same tissue type, this may result in spatial blurring and artificially looking images. Four-dimensional noise reduction using a similarity algorithm (4-dimensional similarity filter, 4D-SF) was recently introduced and can be combined with IR. This algorithm uses per-voxel similarity within the 4D acquisition sequence to average identically perfused tissues, which show similar time-density curves, to reduce image noise. Its application results in a more natural texture depiction with sharper tissue contours that what is obtained with conventional local spatial filters such as the smoothing filter. It also provides very stable images of anatomic structures across the whole dynamic acquisition. The average noise is also generally assumed to be zero, and 4D-SF should lead to no or minimal systematic errors of the time attenuation curves and CT attenuation values [14].

Some studies have reported that 4D-SF can improve image quality for dynamic myocardial computed tomography perfusions (CTPs) and reduce the radiation dose [14, 15]. The noise reduction effect of 4D-SF may be useful for low-dose dynamic breast CT and accurate evaluation of the extent of breast cancer. However, the advantages of low-dose dynamic CT with 4D-SF for breast cancer evaluation have not yet been investigated. This study aimed to evaluate the effects of 4D-SF on the image quality and tumor visibility of low-dose dynamic CT for assessing breast cancer.

## Materials and methods

### Patient selection

This study was approved by our institutional review board. The requirement for informed consent was waived due to the retrospective nature of the study. This study included patients who underwent breast dynamic CT at our hospital between January 2019 and August 2022 for preoperative evaluation and were diagnosed with breast cancer based on surgical pathology specimens. Patients who received neoadjuvant chemotherapy before surgery and those with breast cancers that were difficult to detect or evaluate for extent on CT images were excluded.

### CT technique

**Fig. 1:**
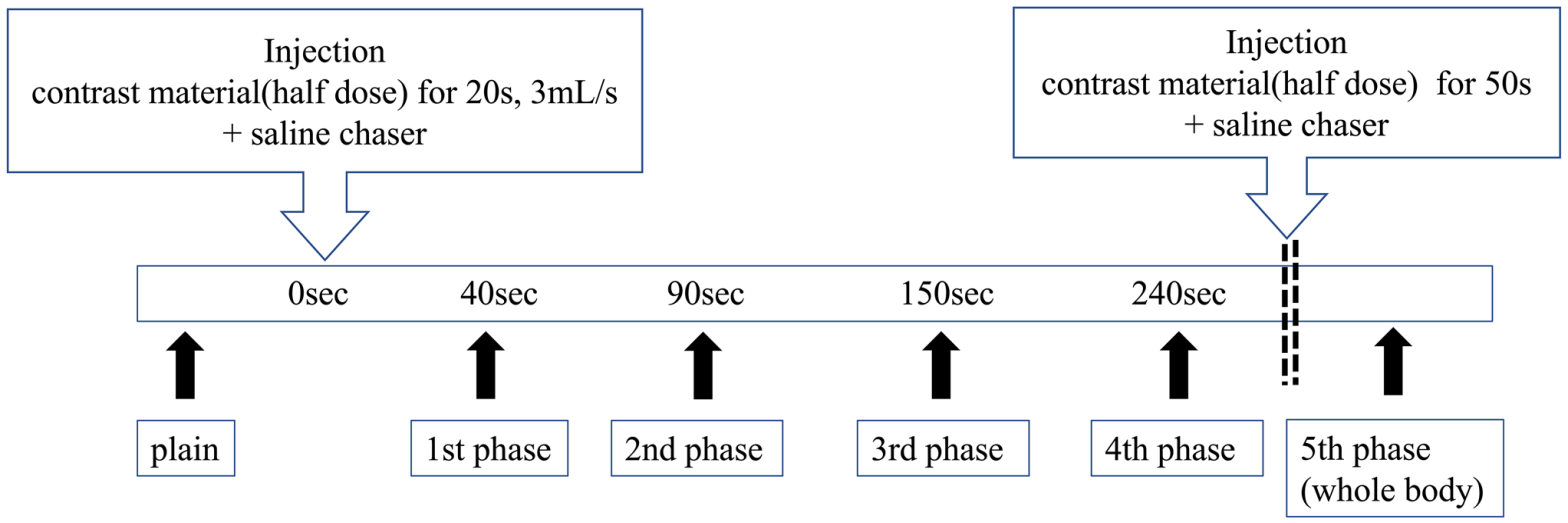
Scanning methods and contrast agent injection for breast dynamic CT

All patients were examined in the supine position using a 320 multidetector CT (Aquilion ONE GENESIS Edition; Canon Medical Systems Inc., Otawara, Japan). The dose of contrast material was determined based on body weight (600 mgI/kg). The radiologist and radiological technologist confirmed the location of breast cancer based on electronic medical records. Subsequently, the scan range of breast dynamic CT was adjusted to include breast cancer with the nipple as a guide in the scout view. Pre-contrast CT images were obtained for dynamic CT. The contrast agent was administered via the antecubital vein through a 22-gauge intravenous catheter with an automatic dual injector (Stellant Dual Flow; Nihon Medrad KK, Osaka, Japan). Non-ionic iodinated contrast agent (iopamidol, 370 mg iodine/mL; Bayer Yakuhin, LTD, Osaka, Japan) and saline were mixed in infusion tube. For dynamic breast CT scans, all patients received 3 mL per second of contrast material and saline over a fixed duration of 20 s, followed by a saline chaser (3.0 mL/s for 10 s). After injecting a half dose of the contrast material, 4-phasic breast dynamic scans were obtained at 40, 90, 150, and 240 s. After 4-phasic breast dynamic scanning, the remaining half dose of contrast material was injected for 50 s, and 20 ml saline was injected at 2 mL/s. Scanning of the last phase was performed after the second injection of contrast material and saline. The last phase covered the entire body for cancer staging, while the other phases for dynamic breast CT covered only the entire breast to reduce radiation exposure and prevent the displacement of the breast lesion resulting from table movements due to the whole-body CT scan. The 4D-SF requires isophasic dynamic volume data without table movements. Figure 1 shows the schema for the scanning methods and contrast agent injection.

The scan parameters for breast dynamic CT were as follows: tube current, 100 mA; tube voltage, 80 kVp; detector collimation, 320 × 0.5 mm; field of view (FOV), 350 mm; and rotation time, 0.5 s/rot. The images in the breast dynamic CT datasets were reconstructed with a 1.0-mm slice thickness using the IR technique (adaptive iterative dose reduction 3D enhanced: AIDR 3D enhanced, Mild) combined with motion compensation based on a non-rigid registration algorithm. The image filtering with 4D-SF was performed as post-processing with a workstation (Vitrea, Canon Medical Systems Inc., Japan). Two types of CT images were acquired. The first was acquired using only the IR technique without the filter processing (4D-SF-), while the other was acquired using a combination of IR and 4D-SF (4D-SF +).

### Image analysis

Of the 4-phasic breast dynamic CT images, only the peak enhancement phase images of breast cancer in each patient were used for further evaluation. For each breast cancer, a circular region of interest (ROI) was placed centrally in the area of the breast cancer (ROI_tumor_, mean size: 40.38 mm^2^). The peak enhancement phase is defined as the phase with the highest CT value for each breast lesion and the most clearly depicted breast lesion for all the breast dynamic CT scan phases. A radiologist with six years of experience in breast imaging (Observer 1) determined the peak enhancement phase using the time–enhancement curve generated based on the attenuation values of breast cancer in Hounsfield units (HU_tumor_) and visual evaluation.

For the quantitative evaluation of image quality, the attenuation values of normal mammary glands (HU_normal_) and standard deviations of attenuation for breast cancer (Noise_tumor_) and the pectoralis major muscle (Noise_muscle_) were measured. The HU_normal_ and Noise_muscle_ were measured from the ROIs placed in the normal mammary glands (ROI _normal_, mean size: 43.75 mm^2^) and the pectoralis major muscle (ROI_muscle_, mean size: 37.51 mm^2^); they were placed in the same slice and as close to the breast cancer and as large as possible. Noise_tumor_ was measured from the ROI_tumor._ The standard deviations of attenuation for the breast cancer (Noise_tumor_) and pectoralis major muscle (Noise_muscle_) were used as image noise. The CT attenuation values and noise were measured using a workstation (ZIO Station 2 version 2.4.2.0a; Ziosoft Inc., Tokyo, Japan). All ROIs placed in 4D-SF images were copied and pasted in 4D-SF + images and placed by Observer 1.

We calculated the signal-to-noise ratio (SNR) and contrast-to-noise ratio (CNR) as follows:

$${\text{SNR}} = {\raise0.7ex\hbox{${{\text{HU}}_{{{\text{tumor}}}} }$} \!\mathord{\left/ {\vphantom {{{\text{HU}}_{{{\text{tumor}}}} } {{\text{Noise}}_{{{\text{tumor}}}} }}}\right.\kern-0pt} \!\lower0.7ex\hbox{${{\text{Noise}}_{{{\text{tumor}}}} }$}}$$

$${\text{CNR}} = \frac{{{\text{HU}}_{{{\text{tumor}}}} - {\text{HU}}_{{{\text{normal}}}} }}{{{\text{Noise}}_{{{\text{muscle}}}} }}$$

The SNRs and CNRs of the 4D-SF- and 4D-SF + images were compared.

Qualitative evaluation of image quality of the 4D-SF- and 4D-SF + images was independently performed by two observers (Observer 1 and Observer 2, who is radiologist with 13 years of experience in breast imaging). The images were evaluated in random order, and the observers were allowed to manually adjust the window width and level, as desired.

Two observers scored each image series for overall image quality, tumor contrast, and tumor margin sharpness using a 4-point scale. The scale used for overall image quality was as follows: 4, no visible image noise or artifacts, sharp anatomic structure, and satisfactory details; 3, mild image noise and artifacts with less clear anatomic structure and details; 2, moderate image noise and artifacts, decreased confidence in details; and 1, severe image noise and artifacts with difficulty in detailed diagnosis. The scale used for tumor contrast was as follows: 4, excellent tumor contrast, clearly visible; 3, good tumor contrast, definitely visible, but lower tumor contrast than 4; 2, reduced tumor contrast and unclear in a part of the tumor; and 1, poor tumor contrast and non-identifiable tumor. The scale for the tumor margin sharpness was as follows: 4, clear margin of entire tumor; 3, visible entire tumor margin, but less clear than 4; 2, unclear margin in a part of the tumor; and 1, unclear margin in most of the tumor.

In addition to Observers 1 and 2, a pathologist (Observer 3) reviewed the CT images and measured the maximum diameters of the breast cancer in the 4D-SF- (size-4D-SF-) and 4D-SF + (size-4D-SF +) images and pathological specimens (size-PS) by consensus. Observer 3 has 17 years of experience in pathology, is a specialist and consultant pathologist, and is an expert in pathological diagnosis. While not directly involved in diagnostic imaging, Observer 3 has experience in comparing pathological and MR images at clinical imaging pathology conferences for breast cancer.

**Fig. 2:**
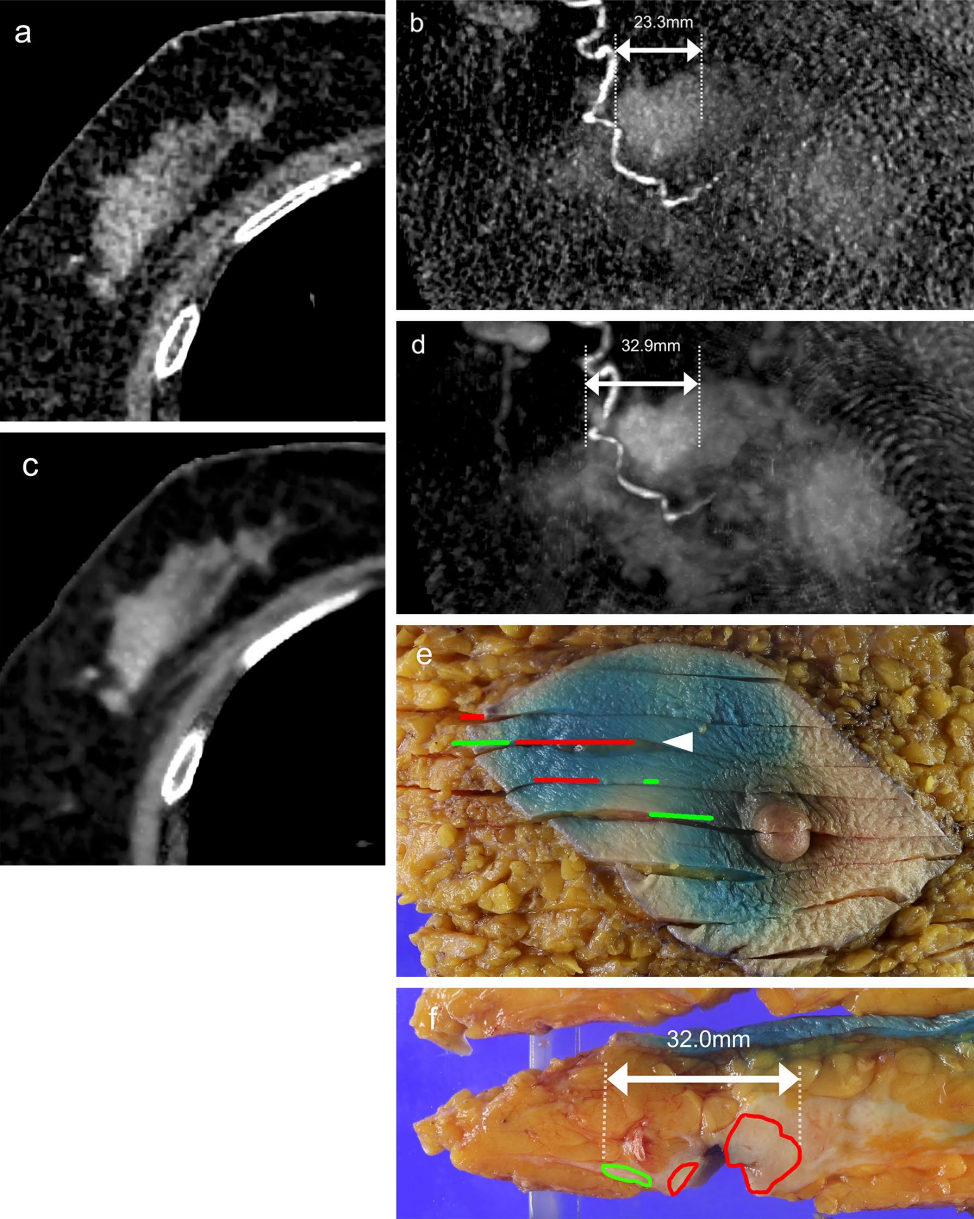
A 74-year-old woman with invasive ductal carcinoma with intraductal extension in the right breast (Group I). Axial CT images and maximum intensity projection (MIP) images acquired without (**a**, **b**) and with (**c**, **d**) 4D-SF showed an enhanced irregular mass and small linear enhancement, which was diagnosed as an intraductal extension. The size-4D-SF- is 23.3 mm, and the size-4D-SF + is 32.9 mm. The size-PS was 32.0 mm in the pathological slice (**f**), which was measured at the arrowhead in the histopathologic map (**e**). The red line corresponds to invasive carcinoma, and the green line corresponds to the intraductal component in the histopathologic map. 4D*-SF* four-dimensional noise reduction filtering using a similarity algorithm, *PS* pathological specimen

**Fig. 3:**
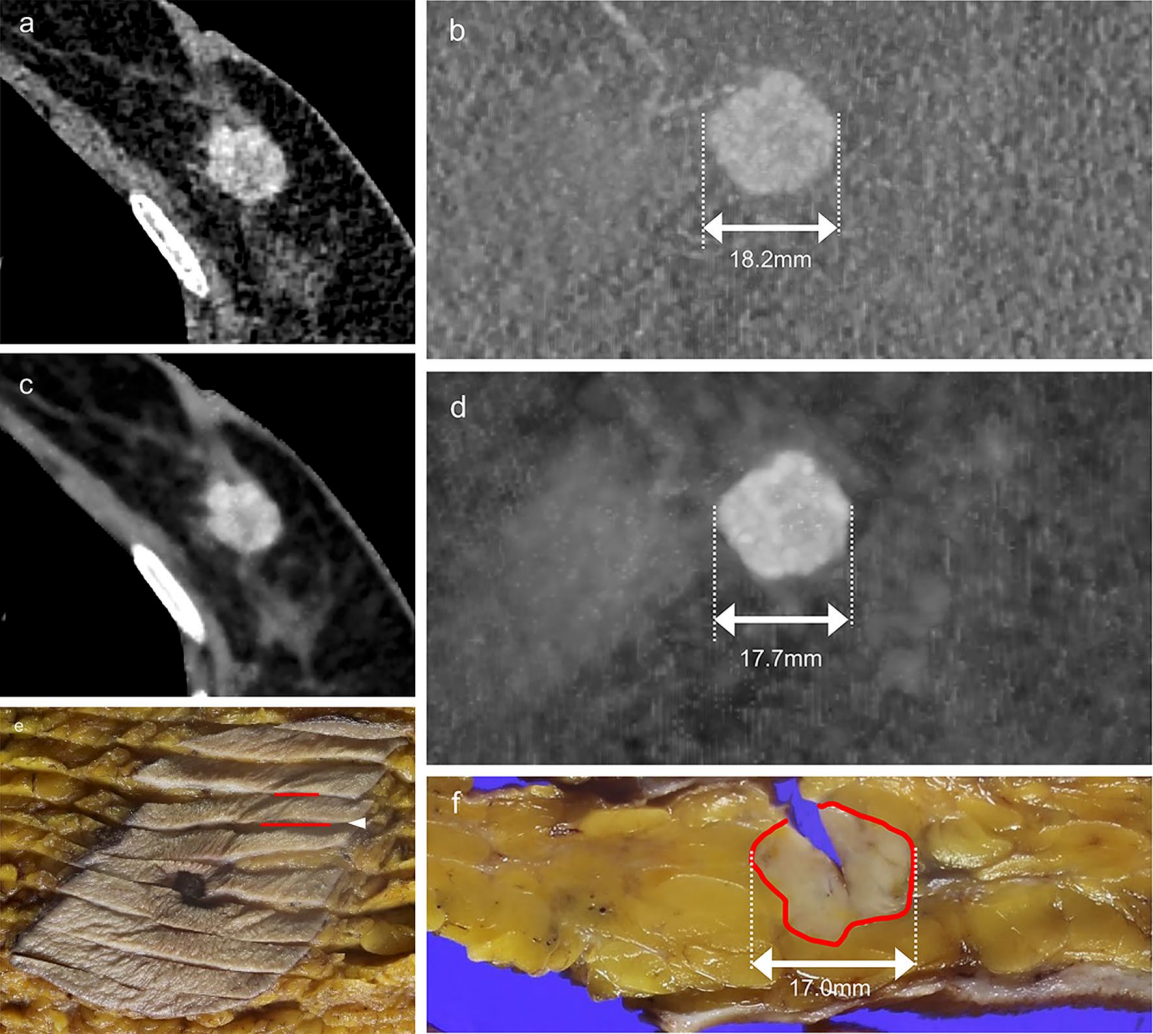
A 72-year-old woman with invasive ductal carcinoma without intraductal extension in the left breast (Group II). Axial CT images and maximum intensity projection (MIP) images acquired without 4D-SF (**a**, **b**) and with 4D-SF (**c**, **d**) showed an enhanced irregular mass. The size-4D-SF- is 18.2 mm, and the size-4D-SF + is 17.7 mm. Size-PS is 17.0 mm in the pathological slice (**f**), which was measured at the arrowhead in the histopathologic map (**e**). The red line corresponds to invasive carcinoma in the histopathologic map. *4D-SF* four-dimensional noise reduction filtering using a similarity algorithm, *PS* pathological specimen

The size-4D-SF- and size-4D-SF + were evaluated at different times with an interval of at least 1 month. The CT images were interpreted with a brief clinical history and knowledge of the histopathologic findings but without knowledge of the findings of other imaging modalities, including mammography, ultrasonography, and MRI. The size-4D-SF- and size-4D-SF + were measured on a dedicated workstation (VINCENT; Fujifilm Medical co., LTD, Tokyo, Japan) in alignment with the orientation of the pathological specimen cut using multiplanar reconstruction and maximum intensity projection images. The correlation between the duration from the CT scan to surgery and the difference between the CT image-derived tumor size and the pathological tumor size were determined. Surgeons at our institution use a surgical margin that is 15 mm beyond the tumor margin, as determined using multimodal imaging. A radiological tumor size underestimated by ≥ 15 mm relative to the pathological tumor size may represent a positive surgical margin. Therefore, we determined the number of lesions with such underestimation detected by 4DSF- and 4DSF + . All enrolled patients with breast cancer were divided into two groups: those with (Group I; Fig. 2) and those without (Group II; Fig. 3) intraductal components. Group I included patients with ductal carcinoma in situ (DCIS) and invasive breast carcinoma with intraductal extension. Group II included patients with invasive breast carcinoma without intraductal extension. The size-4D-SF-, size-4D-SF + , and size-PS were compared for all enrolled breast cancers. These sizes were also compared for groups I and II.

### Pathological assessment

Histological interpretations were made independently by two pathologists including Observer 3. In the event of disagreement, a consensus was reached through discussion. Sectioning of the resected specimens into 5 mm slices was performed according to the General Rules for Clinical and Pathological Recording of Breast Cancer (18th edition) established by the Japanese Breast Cancer Society. The levels of estrogen receptor (ER), progesterone receptor (PR), human epidermal growth factor receptor 2 (HER2), and Ki-67 expressions in patients with invasive breast cancer were determined using streptavidin–peroxidase immunohistochemistry (IHC). A sample was scored as ER- and/or PR-positive when at least 1% of the tumor cell nuclei stained positive for ER or PR, respectively. HER2 expression was evaluated using membrane staining of invasive tumor cells and scored from 0 to 3. HER2 positivity was defined as an IHC HER2 score of 3 or gene amplification by fluorescence in situ hybridization in tumors with an IHC HER2 score of 2 + . The characteristics of the lesions are summarized in Table 1.

**Table 1:** Characteristics of the 38 lesions in this study

Tumor location (right/left)	21/17
Pathological T-stage	
Tis/T1a/T1b/T1c/T2	10/5/4/12/7
Histopathological diagnosis	
IDC	23
ILC	1
Apocrine carcinoma	1
Mucinous carcinoma	1
Mixed carcinoma	
Mucinous and IDC	1
Apocrine and IDC	1
Subtype	
Luminal	21
HER2	1
Triple-negative	6

*IDC* invasive ductal carcinoma, *ILC* Invasive lobular carcinoma, *HER2* human epidermal growth factor receptor

### Statistical analysis

All statistical analyses were performed using the IBM SPSS Statistics version 28.0 (Statistical Package for Social Sciences). Statistical significance was set at p < 0.05. The normality of the data distribution was evaluated using the Kolmogorov–Smirnov test. Continuous measures were summarized as medians and interquartile ranges. The image quality measurements for quantitative evaluation and the overall image quality, tumor contrast, and tumor margin sharpness scores for qualitative evaluation were compared for the 4D-SF- and 4D-SF + images using the Wilcoxon signed-rank test. The Cohen weighted kappa value was used to assess the inter-observer agreement for the evaluation of image quality (> 0.81, excellent agreement; 0.61–0.80, good agreement; 0.41–0.60, moderate agreement; 0.21–0.40, fair agreement; and < 0.20, poor agreement). The maximum diameter of the breast cancer was measured from each CT image, and pathological specimens were compared using the Friedman test with Bonferroni correction for multiple comparisons. The Spearman rank correlation coefficient was used to determine the correlation between the maximum breast cancer diameters measured in the CT images and pathological specimens. The Spearman rank correlation coefficients were classified into no or negligible correlation (Spearman r = 0.01–0.20), low correlation (Spearman r = 0.20–0.40), moderate relation (Spearman r = 0.40–0.70), high correlation (Spearman r = 0.70–0.90), and very high correlation (Spearman r = 0.90–1.00) [16].

## Results

### Patients

Fifty-one patients with 55 lesions underwent breast dynamic CT at our hospital between January 2019 and August 2022 for preoperative evaluation and were diagnosed with breast cancer based on their surgical pathology specimens. Eleven (11 lesions) were excluded for receiving neoadjuvant chemotherapy before surgery. Six (6 lesions) were excluded for breast cancers that were difficult to detect or evaluate for extent on CT images: 1 (1 lesion) underwent biopsy before surgery and had most of the lesion removed; 4 had lesions (4 lesions) that were difficult to distinguish from coexisting benign lesions (such as mastopathy or intraductal papilloma) and background breast enhancement on CT images; and 1 had a lesion localized to the wall of the intracystic carcinoma, and its extent was unknown on CT images. Finally, 34 patients [all female; mean age, 69 years (range 37–92 years)] with 38 lesions were enrolled in the study. Four of them had 2 (instead of 1) lesions: 2 had 2 lesions in the left breast, 1 had 2 lesions in the right breast, and 1 had a lesion in each breast. None of the enrolled lesions had positive surgical margins.

### CT dosage and interval

The mean dose–length product of one phase of breast dynamic CT was 9.9 mGy cm; the estimated effective dose was 0.14 mSv. The estimated effective doses for each scan were calculated by multiplying the dose–length product by a conversion factor of 0.0145 mSv/mGy cm for the chest. The median interval between CT and surgery was 40.5 days (range:13–82 days).

### Quantitative evaluation of image quality

**Fig. 4:**
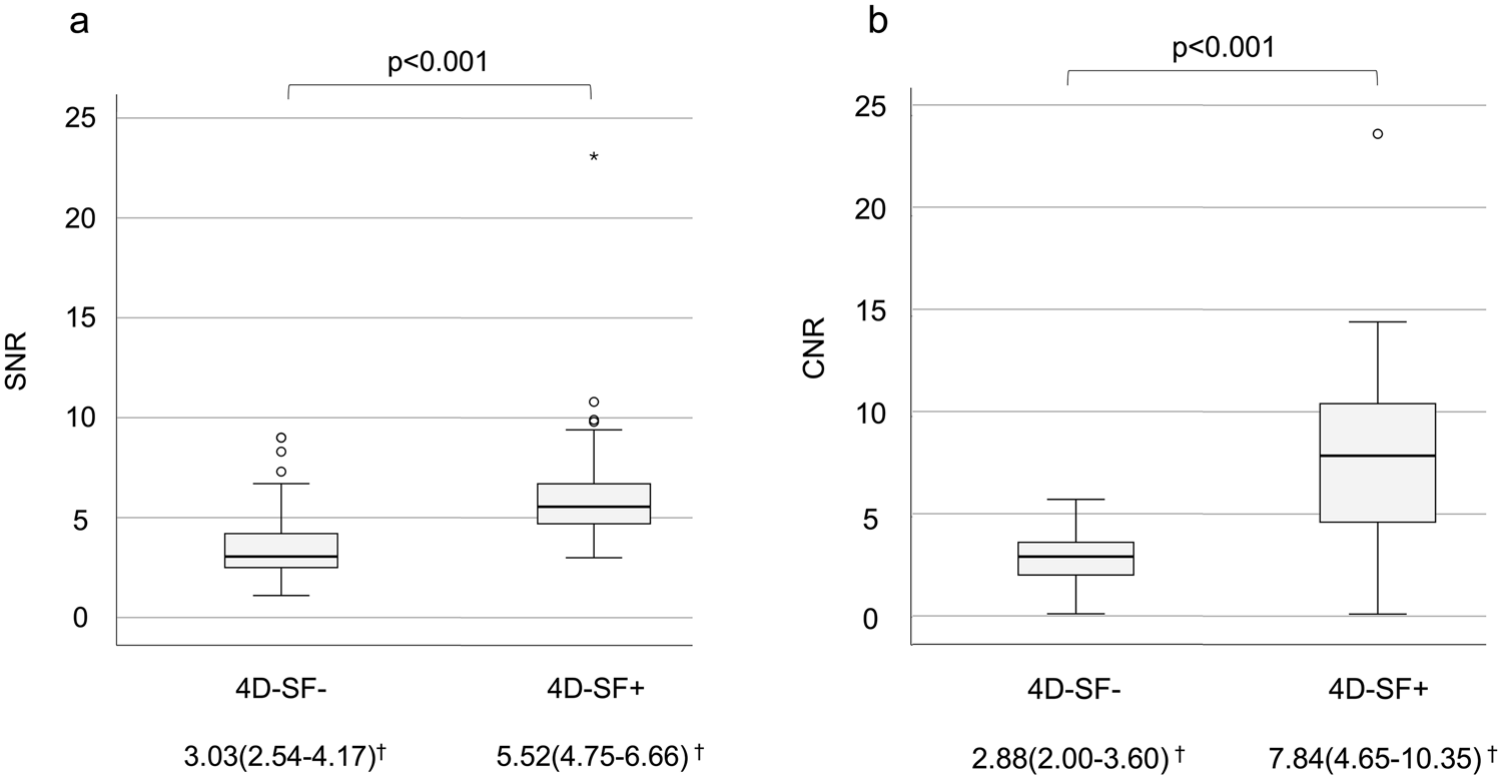
Comparison of the SNRs (**a**) and CNRs (**b**) of the 4D-SF- and 4D-SF + images. ^†^The data are presented as median (first quartile–third quartile). *SNR* signal-to-noise ratio, *CNR* contrast-to-noise ratio, and *4D-SF* four-dimensional noise reduction filtering using a similarity algorithm

The median and interquartile ranges of SNR (4D-SF-: 3.03 [2.54–4.17] vs 4D-SF + : 5.52 [4.75–6.66]) and CNR (4D-SF-: 2.88 [2.00–3.60] vs 4D-SF + : 7.84 [4.65–10.35]) were significantly higher for the 4D-SF + than for the 4D-SF- images (p < 0.001) (Fig. 4a, b).

### Qualitative evaluation of image quality

The median and interquartile range of the overall image quality (Observer 1, p < 0.001; Observer 2, p < 0.001) and tumor margin sharpness (Observer 1, p = 0.003; Observer 2, p < 0.001) scores were significantly higher for 4D-SF + than for 4D-SF-. There was no significant difference between the tumor contrast scores (Observer 1, p = 0.083; Observer 2, p = 0.083) for 4D-SF + and 4D-SF-. Table 2 provides an overview of the results of the qualitative image analysis.

**Table 2:** Qualitative evaluation

	Overall image quality		Tumor contrast		Tumor margin sharpness	
	4D-SF–	4D-SF +	4D-SF–	4D-SF +	4D-SF–	4D-SF +
Observer 1						
Median	1.00 (1, 2)	4.00 (4, 4)*	3.00 (2, 4)	3.00 (2, 4)	3.00 (2, 4)	3.00 (2, 4)*
Observer 2						
Median	1.00 (1, 2)	4.00 (4, 4)*	3.00 (2, 3)	3.00 (2, 3)	3.00 (2, 4)	3.00 (3, 4)*
Κappa	0.73	0.68	0.87	0.86	0.8	0.82

*4D-SF* four-dimensional noise reduction filtering using a similarity algorithm*,* the data are shown as the median (interquartile range)

*The data are significantly higher than those on 4D-SF-

### Correlation of the maximum diameter of breast cancer measured in CT images and pathological specimen

#### All enrolled breast cancers

**Fig. 5:**
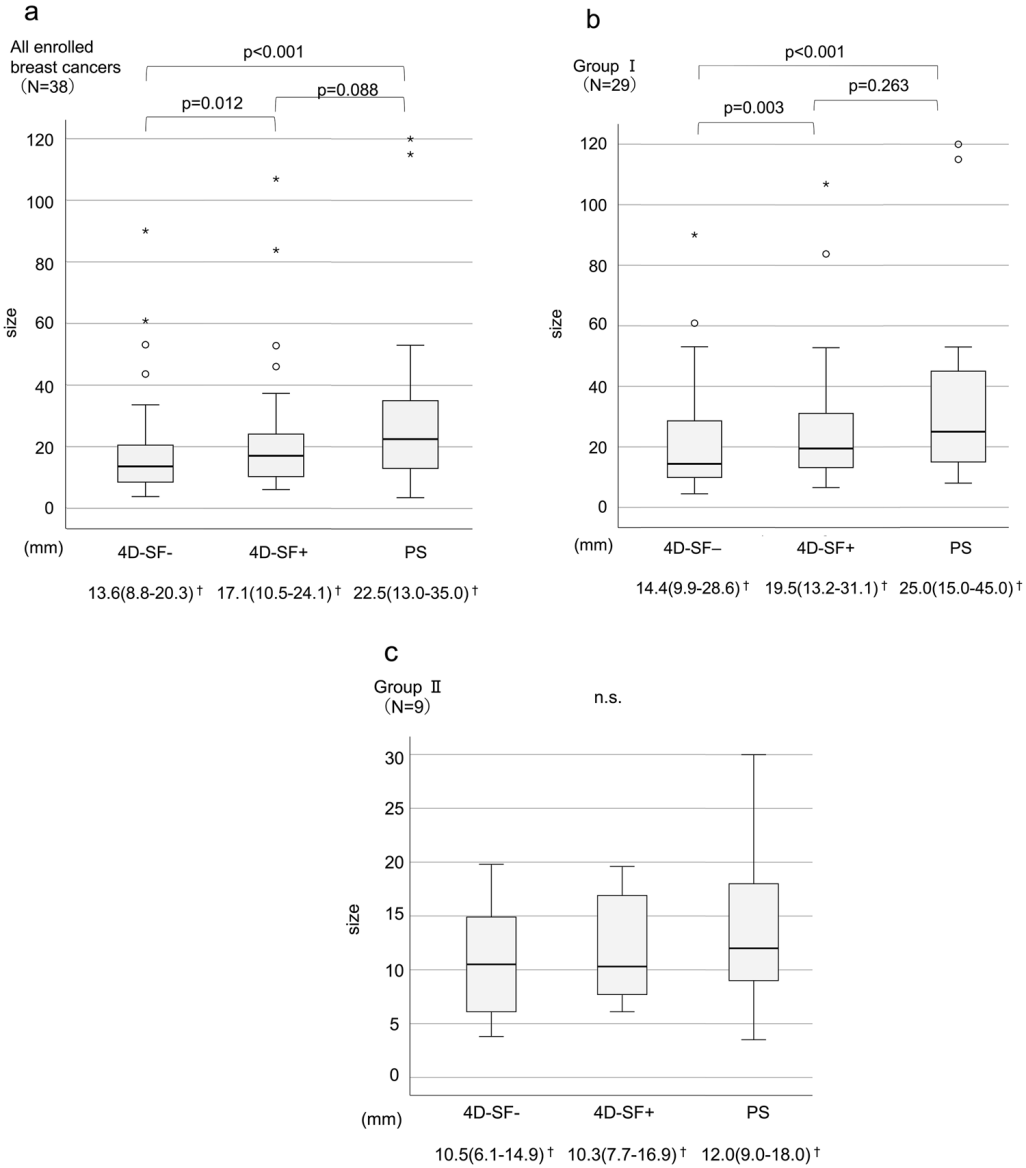
Comparison of the lesion sizes measured from 4D-SF- and 4D-SF + images and pathological specimens of all enrolled breast cancers (**a**), Group I (**b**), and Group II (**c**). ^†^The data are presented as median (first quartile–third quartile). *4D-SF* four-dimensional noise reduction filtering using a similarity algorithm, *n.s.* not significant

**Fig. 6:**
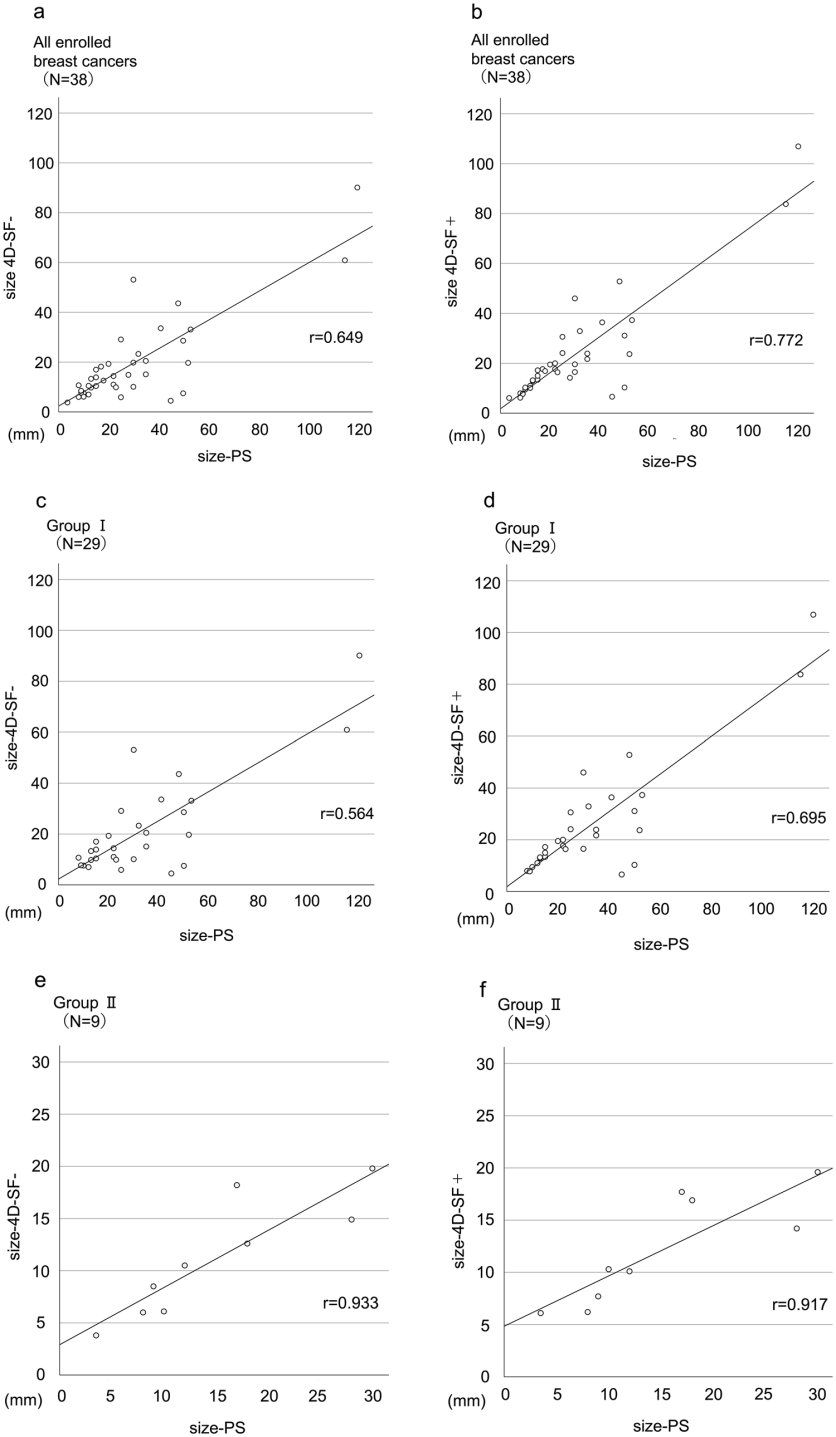
Diagrams of correlations of size-4D-SF-, size-4D-SF + , and size-PS for all enrolled breast cancers (**a**, **b**), Group I (**c**, **d**), and Group II (**e**, **f**) determined with Spearman’s rank correlation analysis. *4D-SF* four-dimensional noise reduction filtering using a similarity algorithm, *PS* pathological specimen

The medians and interquartile ranges of size-4D-SF-, size-4D-SF + , and size-PS for all enrolled breast cancers were 13.6 (8.8–20.3), 17.1 (10.5–24.1), and 22.5 (13.0–35.0) mm, respectively. Size-4D-SF- was significantly smaller than size-4D-SF + (p = 0.012). Size-4D-SF- was significantly smaller than size-PS (p < 0.001); size-4D-SF + was also smaller than size-PS, but the difference was not significant (p = 0.088) (Fig. 5a). There was a significantly moderate correlation between size-PS and size-4D-SF- (r = 0.649, p < 0.001) (Fig. 6a) but a significantly high correlation between size-PS and size-4D-SF + (r = 0.772, p < 0.001) (Fig. 6b).

The duration from the CT scan to surgery and the difference between size-4D-SF + and size-PS (r = 0.303, p = 0.064) were not correlated, but the duration from the CT scan to surgery and the difference between size-4D-SF- and size-PS showed a significantly low correlation (r = 0.330, p = 0.043).

The sizes of 10 breast cancers (2 DCIS, 8 invasive breast cancers with intraductal extension) were underestimated by ≥ 15 mm via evaluation using CT images relative to the pathological tumor size. Of these, the sizes of 6 were underestimated by ≥ 15 mm by both 4D-SF- and 4D-SF + , while those of 4 were underestimated by ≥ 15 mm by only 4D-SF-. No lesion sizes were underestimated by ≥ 15 mm by only 4D-SF + .

Of the 38 breast cancers enrolled, 29 were categorized to Group I (including 10 DCIS and 19 invasive breast carcinomas with intraductal extension) and 9 were categorized to Group II (lesions were invasive breast carcinomas without intraductal extension).

#### Group I (DCIS and invasive breast carcinoma with intraductal extension)

The median and interquartile ranges of size-4D-SF-, size- 4D-SF + , and size-PS were 14.4 (9.9–28.6), 19.5 (13.2–31.1), and 25.0 (15.0–45.0) mm, respectively. Size-4D-SF- was significantly smaller than size-4D-SF + (p = 0.003). Size-4D-SF- was significantly smaller than size-PS (p < 0.001); size-4D-SF + was smaller than size-PS, but the difference was not significant (p = 0.263) (Fig. 5b). There was a significantly moderate correlation between size-PS and size-4D-SF + (r = 0.695, p < 0.001) (Fig. 6d). There was also a significantly moderate correlation between size-PS and size-4D-SF- (r = 0.564, p = 0.001) (Fig. 6c), but it was weaker than that between size-PS and size-4D-SF + .

#### Group II (invasive breast carcinoma without intraductal extension)

The median and interquartile ranges of size-4D-SF-, size- 4D-SF + , and size-PS were 10.5 (6.1–14.9), 10.3 (7.7–16.9), and 12.0 (9.0–18.0) mm, respectively. No significant differences were observed between size-4D-SF-, size-4D-SF + , and size-PS (p = 0.264) (Fig. 5c). There were significantly very high correlations between size-PS and size-4D-SF- (r = 0.933, p < 0.001) (Fig. 6e), and between size-PS and size-4D-SF + (r = 0.917, p < 0.001) (Fig. 6f).

## Discussion

Our quantitative and qualitative evaluations revealed that 4D-SF can significantly improve image quality, which is consistent with previous reports [14, 15]. The evaluation of all enrolled lesions and those in Group I, including DCIS and invasive breast carcinoma with intraductal extension, showed that size-4D-SF- was significantly smaller than size-PS. Size-4D-SF + was also smaller than size-PS but the difference was not significant. Size-4D-SF- showed a significant correlation with size-PS; however, its correlation with size-PS was weaker than that with size-4D-SF + . In contrast, no significant differences were observed between size-4D-SF-, size-4D-SF + , and size-PS of the Group II lesions, including invasive breast carcinoma without intraductal extension. The linear correlations between size-PS and size-4D-SF- and between size-PS and size-4D-SF + were very high for Group II. These results show that using 4D-SF may allow for a more precise delineation of tumor boundaries, especially the intraductal component, by reducing noise. It may facilitate accurate size assessment using low-dose dynamic breast CT. In this study, the sizes of 10 breast cancers (2 DCIS, 8 invasive breast cancers with intraductal extension) were underestimated by ≥ 15 mm via evaluation using CT images relative to the pathological tumor size. This result suggests that the accurate identification of the intraductal components of breast cancer may be difficult using CT images. The underestimation of tumor size by ≥ 15 mm was less frequent with 4D-SF + than with 4D-SF-. We propose that 4D-SF may be useful for accurately evaluating the size of breast cancer with intraductal components.

In this study, the effective dose for the pre-contrast and 4-phasic dynamic CT of the mammary gland was 0.7 mSv. This is comparable to the 0.4 mSv for mammography [17] and lower than previous values reported for the diagnosis of breast cancer using dynamic CT with multidetector CT (the effective dose for pre-contrast and 3-phasic dynamic CT of mammary gland in previous reports; 3.1 mSv [12] and 32.4 mSv [13]). The radiation exposure for lungs with low-dose CT and ultra-low-dose CT, which have been introduced with recent advances in CT technology, are 1–4 mSv and < 1 mSv, respectively [18, 19]. Dynamic breast CT was performed with an extremely low radiation dose in our study. In this study, 4-phasic breast dynamic scans were performed for each patient to obtain the peak enhancement phase images of breast cancer. Considering the radiation exposure, a reduction in the scan phase is desirable. However, only a few studies have investigated the optimal delay for CT scanning after contrast agent injection, and the peak enhancement time for breast cancer varies across reports [10–13]. Moreover, the contrast enhancement pattern is related to the type of pathology [10, 11, 20]. Therefore, the optimal scan timing for breast cancer was not determined in our study, and the number of scan phases could not be reduced.

The IR technique is effective for reducing the radiation dose without impairing image quality [21, 22]. However, IR lacks temporal regularization and has an oversmoothing effect associated with the left shift of the spatial frequency curves toward lower frequencies when the radiation dose is excessively reduced [23]. Other specific techniques are required to extremely reduce the radiation dose while maintaining image quality. Some recent studies have reported that 4D-SF can be used with IR to improve image quality for myocardial CTP. This was useful for radiation dose reduction for myocardial CTP without degrading image quality and affecting the quantification of hemodynamic parameters such as CT-derived myocardial blood flow value [14, 15]. Therefore, we applied 4D-SF to dynamic breast CT, which is used in the cardiac field. In our study, the radiologist and radiological technologist confirmed the location of breast cancer based on the electronic medical records. The scan range was adjusted to include breast cancer with the nipple as a guide in the scout view before performing breast dynamic CT. Lukas et al. reported that a combination of temporal noise reduction and motion compensation was useful for improving the image quality of dynamic myocardial CTP. It significantly improved the sensitivity of myocardial ischemia detection [24]. Unlike the general smoothing filter, 4D-SF provides noise reduction by using per-voxel similarity within the 4D acquisition sequence to average identically perfused tissues [25]. Therefore, 4D-SF requires isophasic dynamic volume data without table movements, and it is important to adjust the scanner to place the organs or lesions to be examined within the scan range.

In this study, we did not compare 4D-SF with recently developed CT technologies such as deep learning reconstruction, dual-energy CT, and photon-counting detector CT. Therefore, the technology suitable for reducing radiation exposure and improving breast dynamic CT image quality has not been established. However, 4D-SF can be used in combination with deep learning reconstruction and dual energy CT, which may lead to a wider range of applications, radiation exposure reduction, and image quality improvement relative to using each technology alone. Sliwicka et al. reported that the combination of deep learning reconstruction and 4D-SF improved the image quality of dynamic myocardial computed tomography perfusion imaging, possibly enabling dose reduction in dynamic CTP imaging for patients with suspected chronic coronary syndrome [26]. Our search did not yield any reports about the clinical utility of the combination of dual energy CT and 4D-SF, and further studies are needed. Photon-counting detector CT has also been adopted in clinical practice, but its usefulness in breast dynamic CT has not been clarified. It is also not clear whether 4D-SF and photon-counting detector CT can be used in combination. However, these may become clearer as photon counting technologies become more practical and widespread. Moreover, 4D-SF can be used regardless of the performance and specification of CT machine unlike dual-energy CT and photon-counting technologies because they are post-processing techniques. This may be advantageous for 4D-SF.

In our study, whole-body CT to screen for tumor metastases was performed after the half dose of contrast material injection was administered after breast 4-phasic dynamic CT. This was to prevent the displacement of the breast lesion resulting from table movements due to the whole-body CT scan. However, this scan timing of whole-body CT was performed later than usual [10, 27, 28]. The detection of liver metastases is important when screening tumor metastases using whole-body CT scans of patients with breast cancer. Two-phase contrast material injections can maintain liver parenchymal enhancement during the delayed phase for a longer duration than monophasic contrast material injection [29]. We believe that biphasic injections are appropriate for our scan protocol for the pretreatment evaluation of patients with breast cancer. Low-tube voltage single-energy CT scanning provides better image contrast due to an increase in iodine contrast [30, 31]. Some studies on liver dynamic CT and whole-body CT have reported that the scan protocol combining low tube voltage (70 or 80 kVp) single-energy CT and reconstruction techniques such as IR can decrease iodine doses (40–50% contrast material reduction) while maintaining CT values for the organs and image quality relative to conventional imaging (120 kVp, no contrast material reduction) [32–34]. Therefore, the 4-phasic breast dynamic CT was performed after injecting a half dose of contrast material determined based on body weight (600 mgI/kg) using low-tube voltage (80 kVp) to increase the iodine contrast.

Our study has some limitations. First, the patient population was relatively small, which introduced a potential for unintended bias. Further investigations with a larger number of cases are required. Second, the duration from the CT scan to surgery with a maximum of 82 days may be a contributing factor to the difference in tumor size determined using CT and pathology. There was a significantly low correlation between the duration from the CT scan to surgery and the difference between size-4D-SF- and size-PS (r = 0.330, p = 0.043). The interval between the CT scan and surgery is a significant limitation in this study. Third, the optimal dose of contrast material and tube voltage for assessing the extent of breast cancer on dynamic CT were not evaluated in our study. However, the aim of this study was to evaluate the effects of 4D-SF on image quality and tumor visibility, while comparing the image quality and accuracy of breast cancer size assessments serves as a method to achieve this goal. Further studies are needed to determine the optimal dose of contrast material and tube voltage for assessing the extent of breast cancer on dynamic breast CT. Fourth, 4-phasic breast dynamic CT scans were obtained with an extremely low radiation dose. However, the optimal radiation dose reduction has not yet been determined. Radiation exposure is a major limitation of CT scanning. Further studies are needed to clarify the optimal radiation dose reduction for CT to evaluate breast cancer extension without impairing diagnostic image quality. Fifth, we did not compare standard contrast-enhanced CT, MR, and 4D-SF + images. Some enrolled patients did not undergo MRI before surgery. However, the study aimed to evaluate the effects of 4D-SF in evaluating breast cancer, and this could be achieved by comparing 4D-SF + and 4D-SF- images. Comparing 4D-SF + images with standard contrast-enhanced CT and MR images for the visibility of breast cancer extent is important for the clinical use of 4D-SF for breast imaging and assessment, and this is our next research topic. Sixth, the time-density curve was not evaluated because the criteria for defining descriptors in its pattern assessments, such as breast dynamic contrast-enhanced MRI based on Breast Imaging Reporting and Data System (BI-RADS) MRI lexicon, have not been established for breast dynamic CT [35]. Moreover, the optimal timing for dynamic breast CT remains unclear. In our study, dynamic scanning was performed to obtain the peak enhancement phase images of breast cancer. The 4D-SF is mainly used for scanning perfusion CT imaging. It may improve the accuracy of the time density curve and may be useful for accurate pattern evaluation of TDC for distinguishing benign from malignant breast lesions on breast dynamic CT. Further studies are required to clarify the influence of 4D-SF on the time-density curve.

In conclusion, 4D-SF can improve the image quality and tumor visibility of low-dose dynamic CT for the evaluation of breast cancer due to noise reduction. This may improve the accuracy of the evaluations of the extent of breast cancer with intraductal components.

## References

[1] Bray F, Laversanne M, Sung H, Ferlay J, Siegel RL, Soerjomataram I, et al. Global cancer statistics 2022: GLOBOCAN estimates of incidence and mortality worldwide for 36 cancers in 185 countries. CA Cancer J Clin. 2024;74(3):229–63. 38572751 10.3322/caac.21834

[2] Mansfield CM, Komarnicky LT, Schwartz GF, Rosenberg AL, Krishnan L, Jewell WR, et al. Ten-year results in 1070 patients with stages I and II breast cancer treated by conservative surgery and radiation therapy. Cancer. 1995;75(9):2328–36. 7712444 10.1002/1097-0142(19950501)75:9<2328::aid-cncr2820750923>3.0.co;2-l

[3] Nakahara H, Namba K, Wakamatsu H, Watanabe R, Furusawa H, Shirouzu M, et al. Extension of breast cancer: comparison of CT and MRI. Radiat Med. 2002;20(1):17–23. 12002599

[4] Ahn SJ, Kim YS, Kim EY, Park HK, Cho EK, Kim YK, et al. The value of chest CT for prediction of breast tumor size: comparison with pathology measurement. World J Surg Oncol. 2013;11:130. 23741999 10.1186/1477-7819-11-130 PMC3698146

[5] Goto M, Ito H, Akazawa K, Kubota T, Kizu O, Yamada K, et al. Diagnosis of breast tumors by contrast-enhanced MR imaging: comparison between the diagnostic performance of dynamic enhancement patterns and morphologic features. J Magn Reson Imaging. 2007;25(1):104–12. 17152054 10.1002/jmri.20812

[6] Shimauchi A, Yamada T, Sato A, Takase K, Usami S, Ishida T, et al. Comparison of MDCT and MRI for evaluating the intraductal component of breast cancer. AJR Am J Roentgenol. 2006;187(2):322–9. 16861533 10.2214/AJR.05.0876

[7] Uematsu T. Comparison of magnetic resonance imaging and multidetector computed tomography for evaluating intraductal tumor extension of breast cancer. Jpn J Radiol. 2010;28(8):563–70. 20972855 10.1007/s11604-010-0474-5

[8] Morrogh M, Morris EA, Liberman L, Borgen PI, King TA. The predictive value of ductography and magnetic resonance imaging in the management of nipple discharge. Ann Surg Oncol. 2007;14(12):3369–77. 17896158 10.1245/s10434-007-9530-5

[9] Uematsu T, Yuen S, Kasami M, Uchida Y. Comparison of magnetic resonance imaging, multidetector row computed tomography, ultrasonography, and mammography for tumor extension of breast cancer. Breast Cancer Res Treat. 2008;112(3):461–74. 18193352 10.1007/s10549-008-9890-y

[10] Okada K, Matsuda M, Tsuda T, Kido T, Murata A, Nishiyama H, et al. Dual-energy computed tomography for evaluation of breast cancer: value of virtual monoenergetic images reconstructed with a noise-reduced monoenergetic reconstruction algorithm. Jpn J Radiol. 2020;38(2):154–64. 31686294 10.1007/s11604-019-00897-1

[11] Inoue M, Sano T, Watai R, Ashikaga R, Ueda K, Watatani M, et al. Dynamic multidetector CT of breast tumors: diagnostic features and comparison with conventional techniques. AJR Am J Roentgenol. 2003;181(3):679–86. 12933459 10.2214/ajr.181.3.1810679

[12] Seo BK, Pisano ED, Cho KR, Cho PK, Lee JY, Kim SJ. Low-dose multidetector dynamic CT in the breast: preliminary study. Clin Imaging. 2005;29(3):172–8. 15855061 10.1016/j.clinimag.2004.04.029

[13] Kuroki-Suzuki S, Kuroki Y, Ishikawa T, Takeo H, Moriyama N. Diagnosis of breast cancer with multidetector computed tomography: analysis of optimal delay time after contrast media injection. Clin Imaging. 2010;34(1):14–9. 20122514 10.1016/j.clinimag.2009.03.004

[14] Kouchi T, Tanabe Y, Smit EJ, Kido T, Kurata A, Kouchi Y, et al. Clinical application of four-dimensional noise reduction filtering with a similarity algorithm in dynamic myocardial computed tomography perfusion imaging. Int J Cardiovasc Imaging. 2020;36(9):1781–9. 32399762 10.1007/s10554-020-01878-6

[15] Yamamoto Y, Tanabe Y, Kurata A, Yamamoto S, Kido T, Uetani T, et al. Feasibility of four-dimensional similarity filter for radiation dose reduction in dynamic myocardial computed tomography perfusion imaging. Front Radiol. 2023;3:1214521. 38105799 10.3389/fradi.2023.1214521 PMC10722229

[16] Guilford JP. Fundamental statistics in psychology and education. New York: McGraw-Hill Book Company; 1957. p. 219.

[17] Meyer C, Millan P, Gonzalez V, Spera G, Machado A, Mackey JR, et al. Intensive imaging surveillance of survivors of breast cancer may increase risk of radiation-induced malignancy. Clin Breast Cancer. 2019;19(3):e468–74. 30850181 10.1016/j.clbc.2019.01.003

[18] Garg M, Devkota S, Prabhakar N, Debi U, Kaur M, Sehgal IS, et al. Ultra-low dose CT chest in acute COVID-19 pneumonia: a pilot study from India. Diagnostics (Basel). 2023;13(3):351. 36766456 10.3390/diagnostics13030351 PMC9914217

[19] (AAPM) AAoPiM. Lung cancer screening CT protocols version 5.1. 2019.

[20] Harish MG, Konda SD, MacMahon H, Newstead GM. Breast lesions incidentally detected with CT: what the general radiologist needs to know. Radiographics. 2007;27(Suppl 1):S37-51. 18180233 10.1148/rg.27si075510

[21] Gramer BM, Muenzel D, Leber V, von Thaden AK, Feussner H, Schneider A, et al. Impact of iterative reconstruction on CNR and SNR in dynamic myocardial perfusion imaging in an animal model. Eur Radiol. 2012;22(12):2654–61. 22752461 10.1007/s00330-012-2525-z

[22] Bhave NM, Mor-Avi V, Kachenoura N, Freed BH, Vannier M, Dill K, et al. Analysis of myocardial perfusion from vasodilator stress computed tomography: does improvement in image quality by iterative reconstruction lead to improved diagnostic accuracy? J Cardiovasc Comput Tomogr. 2014;8(3):238–45. 24939073 10.1016/j.jcct.2014.04.008

[23] Millon D, Vlassenbroek A, Van Maanen AG, Cambier SE, Coche EE. Low contrast detectability and spatial resolution with model-based Iterative reconstructions of MDCT images: a phantom and cadaveric study. Eur Radiol. 2017;27(3):927–37. 27300195 10.1007/s00330-016-4444-x

[24] Lukas S, Feger S, Rief M, Zimmermann E, Dewey M. Noise reduction and motion elimination in low-dose 4D myocardial computed tomography perfusion (CTP): preliminary clinical evaluation of the ASTRA4D algorithm. Eur Radiol. 2019;29(9):4572–82. 30715584 10.1007/s00330-018-5899-8

[25] Tsuneta S, Oyama-Manabe N, Kameda H, Harada T, Kato F, Smit EJ, et al. Improvement of image quality on low-dose dynamic myocardial perfusion computed tomography with a novel 4-dimensional similarity filter. Medicine (Baltimore). 2020;99(26): e20804. 32590765 10.1097/MD.0000000000020804 PMC7328929

[26] Sliwicka O, Swiderska-Chadaj Z, Snoeren M, Brink M, Salah K, Peters-Bax L, et al. Multireader image quality evaluation of dynamic myocardial computed tomography perfusion imaging with a novel four-dimensional noise reduction filter. Acta Radiol. 2023;64(3):999–1006. 35765201 10.1177/02841851221108804

[27] Noda Y, Kaga T, Kawai N, Miyoshi T, Kawada H, Hyodo F, et al. Low-dose whole-body CT using deep learning image reconstruction: image quality and lesion detection. Br J Radiol. 2021;94(1121):20201329. 33571010 10.1259/bjr.20201329 PMC8506192

[28] Noda Y, Kawai N, Kawamura T, Kobori A, Miyase R, Iwashima K, et al. Radiation and iodine dose reduced thoraco-abdomino-pelvic dual-energy CT at 40 keV reconstructed with deep learning image reconstruction. Br J Radiol. 2022;95(1134):20211163. 35230135 10.1259/bjr.20211163 PMC10996425

[29] Ichikawa T. CT zouei riron (Theory of CT imaging), vol. 54–6. Tokyo: Igaku-Shoin; 2004. p. 178–83.

[30] Nakayama Y, Awai K, Funama Y, Liu D, Nakaura T, Tamura Y, et al. Lower tube voltage reduces contrast material and radiation doses on 16-MDCT aortography. AJR Am J Roentgenol. 2006;187(5):W490–7. 17056879 10.2214/AJR.05.0471

[31] Hisoshima M, Urano M, Ohashi K, Ogawa M, Omata S, Yoshida S, et al. Utility of 70-kV single-energy CT in depicting the extent of breast cancer for preoperative planning. Breast Cancer Res Treat. 2020;184(3):817–23. 32910319 10.1007/s10549-020-05909-7

[32] Ichikawa S, Motosugi U, Shimizu T, Kromrey ML, Aikawa Y, Tamada D, et al. Diagnostic performance and image quality of low-tube voltage and low-contrast medium dose protocol with hybrid iterative reconstruction for hepatic dynamic CT. Br J Radiol. 2021;94(1128):20210601. 34586900 10.1259/bjr.20210601 PMC8631019

[33] Tachibana Y, Takaji R, Shiroo T, Asayama Y. Deep-learning reconstruction with low-contrast media and low-kilovoltage peak for CT of the liver. Clin Radiol. 2024;79(4):e546–53. 38238148 10.1016/j.crad.2023.12.015

[34] Yoshida M, Nakaura T, Oda S, Kidoh M, Nagayama Y, Uetani H, et al. Effects of tube voltage and iodine contrast medium on radiation dose of whole-body CT. Acta Radiol. 2022;63(4):458–66. 33709794 10.1177/02841851211001539

[35] Dorsi C, Sickles E, Mendelson E, Morris E. Breast imaging reporting and data system. 5th ed. Reston: American College of Radiology; 2013.

